# The role of c-kit and imatinib mesylate in uveal melanoma

**DOI:** 10.1186/1477-3163-4-19

**Published:** 2005-10-19

**Authors:** Patricia Rusa Pereira, Alexandre Nakao Odashiro, Jean Claude Marshall, Zelia Maria Correa, Rubens Belfort, Miguel N Burnier

**Affiliations:** 1 Department of Ophthalmology, Federal University of São Paulo, São Paulo, Brazil; 2 Henry C. Witelson Ocular Pathology Laboratory, Department of Ophthalmology, McGill University, Montreal – Canada

**Keywords:** C-kit, CD117, melanoma, uveal, ocular, Gleevec^®^, Imatinib mesylate

## Abstract

**Background:**

Uveal melanoma (UM) is the most common primary intraocular tumor in adults, leading to metastasis in 40% of the cases and ultimately to death in 10 years, despite local and/or systemic treatment. The c-kit protein (CD117) is a membrane-bound tyrosine kinase receptor and its overexpression has been observed in several neoplasms. Imatinib mesylate is a FDA approved compound that inhibits tyrosine quinase receptors, as well as c-kit. Imatinib mesylate controls tumor growth in up to 85% of advanced gastrointestinal stromal tumors, a neoplasia resistant to conventional therapy.

**Methods:**

Fifty-five specimens of primary UM selected from the archives of the Ocular Pathology Laboratory, McGill University, Montreal, Canada, were immunostained for c-kit. All cells displaying distinct immunoreactivity were considered positive. Four human UM cell lines and 1 human uveal transformed melanocyte cell line were tested for i*n vitro *proliferation Assays (TOX-6) and invasion assay with imatinib mesylate (concentration of 10 μM).

**Results:**

The c-kit expression was positive in 78.2% of the UM. There was a statistical significant decrease in the proliferation and invasion rates of all 5 cell lines.

**Conclusion:**

The majority of UM expressed c-kit, and imatinib mesylate does decrease the proliferation and invasion rates of human UM cell lines. These results justify the need for a clinical trial to investigate in vivo the response of UM to imatinib mesylate.

## Background

Uveal melanoma (UM) is the most common primary intra-ocular tumour in adults [1], with an incidence of five to six individuals per million people [2]. Forty percent of UM patients will progress from local to systemic disease developing metastasis that will ultimately lead to death after 10 years of diagnosis, despite the treatment options such as local radiotherapy, enucleation and systemic chemotherapy [3].

C-kit (Kit, CD117, stem cell factor receptor) is a 145 kDa transmembrane tyrosine kinase protein that acts as a type-III receptor. The c-kit proto-oncogene, located on chromosome 4q11-21, encodes the c-kit, which ligand is the stem cell factor (SCF, steel factor, kit ligand, mast cell growth factor) [4,5]. Tyrosine phosphorylation by protein tyrosine kinases is of particular importance in cellular signalling and can mediate signals for major cellular processes, such as proliferation, differentiation, apoptosis, attachment, and migration. The role of c-kit expression has been studied in hematologic and solid tumours, such as acute leukemias [6], and gastrointestinal stromal tumors (GIST) [7].

The clinical importance of c-kit expression in malignant tumors relies on the existence of a compound (imatinib mesylate, STI571, Gleevec^®^, Novartis Pharma AG Basel, Switzerland) that specifically inhibits tyrosine kinase receptors [8]. Moreover, a clinically relevant breakthrough has been the finding of remarkable anti-tumor effects of this compound in GIST, a group of tumors regarded as being generally resistant to conventional chemotherapy [9]. Imatinib mesylate has been approved by the United States-FDA to treat c-kit positive GIST and Philadelphia-chromosome-positive chronic myelogenous leukemia [10]. The purpose of this article is to study the immunoexpression of c-kit in UM, as well as the *in vitro *effects of imatinib mesylate on UM cell lines.

## Materials and methods

### Paraffin blocks

Formalin-fixed, paraffin-embedded blocks from enucleation of primary choroidal melanoma were collected from the archives of the Henry C. Witelson Ocular Pathology Laboratory, McGill University, Montreal, Canada from the years 1980–2004. All the cases have sufficient tumor material for analysis. The tumors with irradiation were excluded.

### Immunohistochemistry

Immunohistochemistry was performed using the polyclonal anti-CD117 antibody A4502 (Dako, Mississauga, Ontario, Canada). The antibody was applied at a dilution of 1:300 for 18 h at 4°C, after 15 minutes in 10 nmol/l citrate buffer (pH 6.0) for antigen retrieval. Endogenous peroxidase was blocked using 0.3% hydrogen peroxidase diluted in methanol for 30 minutes. A standard avidin-biotin complex (ABC) technique using diaminobenzidine was used for visualization, with a red colouring stain to avoid misinterpretation in pigmented tumors. A case of KIT-positive Gastrointestinal Stromal Tumor (GIST) was used as control. Negative control sections were incubated with normal rabbit serum instead of the primary antibody.

After tissue processing, all cells displaying distinct immunoreactivity were considered positive, irrespective of staining intensity. We assigned the results of c-kit staining as negative when no staining was present, low when less than 50%, and high when more than 50% of melanoma cells were positive. The immunoreactivity was categorized as cytoplasmatic or membranous expression using the grade system described above. In order to better characterize the c-kit expression in uveal melanomas, the expression in the different cell types (spindle and epithelioid – modified Callender's classification) was analysed, in the mixed cell, predominant epithelioid and predominant spindle tumors.

### Cell Culture

Four human UM cell lines (92.1, SP6.5, MKT-BR, OCM-1) and one human uveal transformed melanocyte cell line (UW-1) were incubated at 37°C in a humidified 5% CO_2_-enriched atmosphere. The cells were cultured in RPMI-1640 medium (Invitrogen, Burlington, Ontario, Canada), supplemented with 5% heat inactivated fetal bovine serum (FBS), 1% fungizone, and 1% penicillin-streptomycin purchased from Invitrogen (Burlington, Ontario, Canada). Cells were cultured as a monolayer in 25 cm^2 ^flasks (Fisher, Whitby, Ontario, Canada) and observed twice weekly, at every media change, for normal growth by phase contrast microscopy. The cultures were grown to confluence and passage by treatment with 0.05% trypsin in EDTA (Fisher) at 37°C and washed in 7 ml RPMI-1640 media before being centrifuged at 120 g for 10 minutes to form a pellet. Cells were then suspended in 1 ml of medium and counted using the Trypan Blue dye exclusion test.

The UM cell lines 92.1, SP6.5, and MKT-BR were established by Dr. Jager (University Hospital Leiden, The Netherlands), Dr. Pelletier (Laval University, Quebec, Canada), Dr. Belkhou (CJF INSERM, France), respectively. Dr. Albert (University of Wisconsin-Madison, USA) established the OCM-1 and UW-1 cell lines [11,12].

### In Vitro Invasion Assay

A modified Boyden chamber consisting of a polyethelene teraphthalate membrane (PET) with 8-um diameter pores, precoated with Matrigel, an artificial basement membrane, (Beckton Dickenson Labware, Bedford, MA) was used as previously described [13], to assay for invasive ability. PET membrane without Matrigel was used as a control.

Briefly, 1.25 × 10^5 ^cells were added to the upper chamber in RPMI-1640 medium with 0.1% FBS. RPMI-1640 medium with 10% FBS was added to the lower chamber as a chemoattractant to obtain the baseline invasive ability of the cell lines. The effect of imatinib mesylate on invasion was assayed by adding 10 μM of imatinib mesylate to the RPMI-1640 medium supplemented with 10% FBS in the upper chamber. The concentrations of imatinib mesylate was chosen based on the blood levels reported in previous studies with the maximum tolerated dose of imatinib mesylate on clinical trials [14]. The chambers were then incubated at 37°C in a 5% CO_2_-enriched atmosphere for 48 hours to allow for cellular invasion through the Matrigel.

Non-invading cells were removed from the upper chamber by gently wiping the surface of the membrane with a moist cotton swab. Membranes were removed and then stained using a Diff-Quick staining set, which stains cell nuclei purple and cytoplasm pink. Stained cells were counted microscopically in 20 high-powered fields, randomly. Only cells whose nuclei had completely invaded through the membrane were counted. Each experimental condition, including control, was performed in triplicate and the average number of invading cells was then calculated for all experimental conditions.

Percent invasion was determined for each cell line under each experimental condition using the following formula: % invasion = (mean number of cells invading through the matrigel/mean number of cells migrating through control PET membrane) multiplied by a hundred. The cell lines were then ranked according to their invasive ability.

### *In Vitro *Proliferation Assay

The Sulforhodamine-B based assay kit (TOX-6, Sigma-Aldrich) was performed as per the National Cancer Institute protocol [15]. Briefly, five human UM cell lines were seeded into wells at a concentration of 2.5 × 10^3 ^cells per well, in a minimum of six wells per cell line. A row of 8 wells exposed to only RPMI-1640 medium was used as a control. Twenty-four hours following seeding, imatinib mesylate was added to the experimental wells. The concentration of imatinib mesylate was 10 μM [14]. Cells were allowed to incubate for 48 hours following cell seeding. Following this 48 hour period, cells were fixed to the bottom of the wells using a solution of 50% Trichloroacetic acid (TCA) for 1 hour at 4°C. Plates were then rinsed with distilled water, to remove TCA and medium, and air dried. The Sulforhodamine-B dye was added to each well and allowed to stain for 25 minutes. The Sulforhodamine-B dye was subsequently removed by washing with a 10% acetic acid solution and once more allowed to air dry. The dye that was incorporated into the fixed cells at the bottom of the wells was solubilized in a 10 mM solution of Tris. The absorbance of the solute was measured using a microplate reader at a wavelength of 510 nm. This gave a comparison of control cell proliferation rate over 48 hours compared to proliferation rate of cells exposed to imatinib mesylate during the same time period at a dose of 10 μM.

### Statistical Analysis

The differences in invasion rates under three experimental conditions for each uveal melanoma cell line were determined using the ANOVA test. A p value of less than 0.05 was considered statistically significant. Calculations were computer-based (SPSS 11.5, SPSS Inc., Chicago, Illinois, USA).

## Results

Fifty-five cases of UM were studied. Eight seven percent of the tumors (n = 48) were classified as mixed cell type (spindle and epithelioid cells), 9% (n = 5) had predominance of epithelioid cells, and 3.6% (n = 2) of spindle cells.

**Figure 1:**
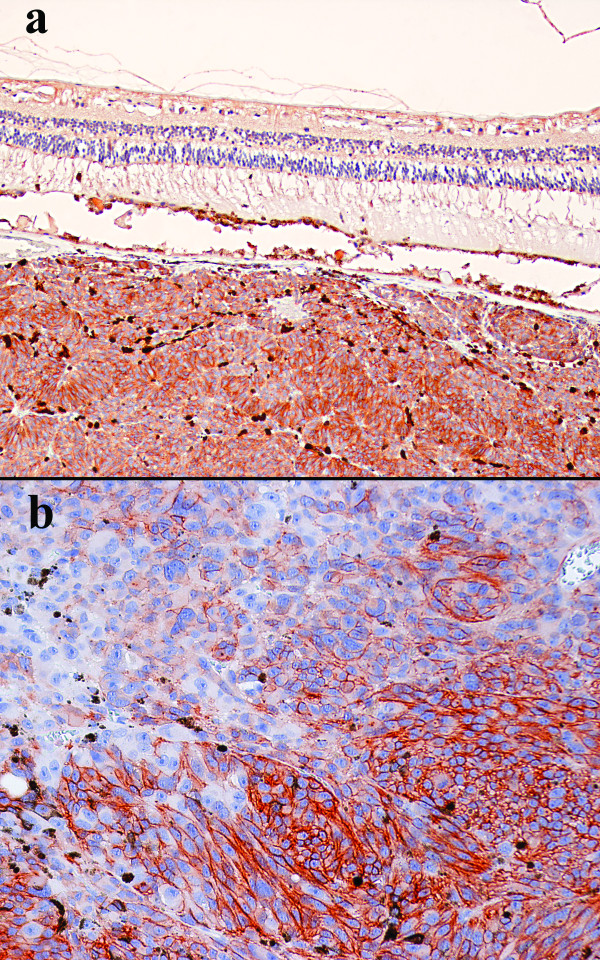
A. Choroidal UM displaying high expression of c-kit. The above retina is somewhat detached. Figure 1B. UM composed of epithelioid cells showing a cell membrane pattern of immunostain.

Seventy-eight percent of cases (n = 43) were found to be c-kit positive (Figure 1A). Among the positive cases, 46.5% (n = 20) presented with what was considered as high expression. All lesions with high immunoreactivity (n = 20) had cytoplasmic and cell membrane expression. Meanwhile, among lesions with low immunoreactivity (n = 23), 100% presented a cytoplasmatic reaction and just 30.4% (n = 7) presented with a cell membrane stain-pattern (Figure 1B). (Table 1)

**Table 1:** High, low and negative expression of c-kit in different cell types and different patterns of stain.

	High expression	Low expression	Negative
Spindle cells	36.8%	57.9%	5.3%
Epithelioid cells	41.5%	53.6%	4.9%
Cell membrane	100%	30.4%	------
Cytoplasmatic	100%	100%	------

**Figure 3:**
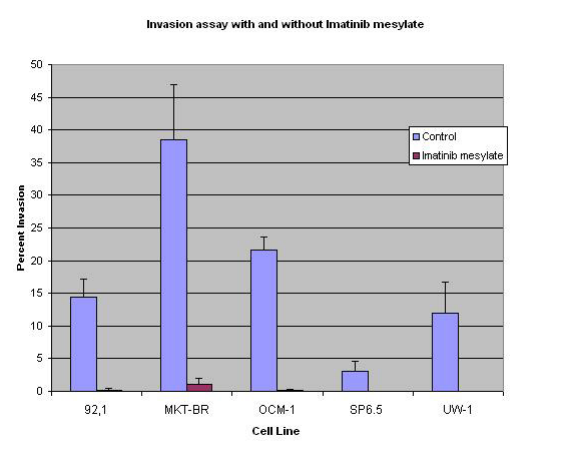
The graph shows the invasion assay results for each cell line with experimental conditions. The blue bar for each represents the control proliferation without Imatinib Mesylate, and the red bar with the compound. A statistical difference was seen for the five cell lines after 10 μM exposure.

The percent invasion of cell lines according to the baseline invasion without imatinib mesylate was: MKT-BR (38.4%) > OCM-1 (21.7%) > 92.1 (14.4%) > UW-1 (12%) > SP6.5 (3%). The addition of imatinib mesylate decreased the invasion in all cell lines: MKT-BR (1.03%); OCM-1 (0.1%); 92.1 (0.2%); UW-1 (0%); SP6.5 (0%). The results are shown in Figure 3.

**Figure 2:**
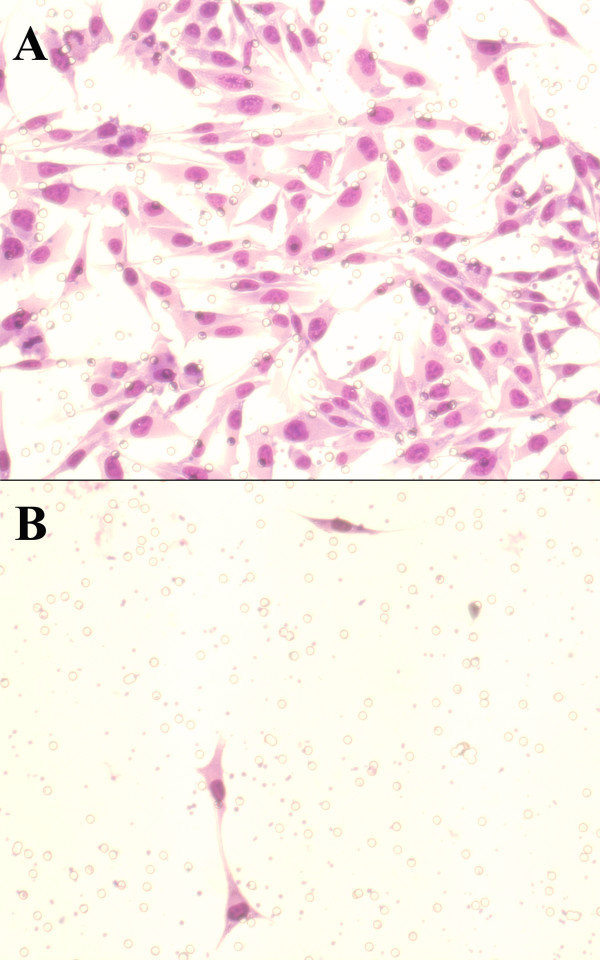
A. Photomicrograph of UM cells without Imatinib mesylate (control) invading through Matrigel (200×). Figure 2B. Photomicrograph of UM cells treated with Imatinib mesylate invading through Matrigel (200×).

No visible changes in cytomorphology were seen in reaction to the presence of imatinib mesylate (figure 2).

Statistically significant differences between the invasion rates for the control group and imatinib mesylate group were found in all cell lines (T test p value < 0.05).

**Figure 4:**
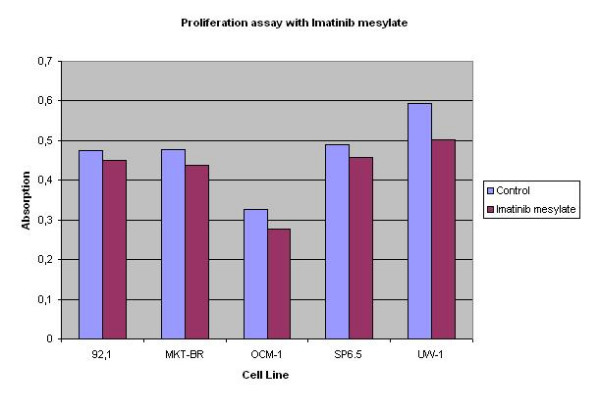
The graph shows the proliferation assay results comparing a control and the Imatinib mesylate exposure cells. A statistical difference was seen for the five cell lines after 10 μM exposure.

Figure 4 depicts the results from the Sulforhodamine-B assay. The mean and standard deviation for each cell line per condition is shown in Table 2. Cells that were directly exposed imatinib mesylate showed a decrease in proliferation for all five human cell lines (92.1, MKT-BR, OCM-1, SP6.5, UW-1) as compared to control (p value of 0.001354991, 0.012655861, 9.47698 × 10^-7^, 0.002754018 and 5.79576 × 10^-6 ^respectively).

**Table 2:** Number of cells counted per control membrane, and percent invasion (mean ± standard deviation) of cells in response to experimental conditions.

**Cell Line**	**Average Number of Cells in Control PET**	**Percent Invasion of Cells in Response to 10% FBS**	**Percent Invasion of Cells in Response to Imatinib mesylate**
MKT-BR	247	38.43% ± 8.5	1.03% ± 0.2
OCM-1	246.67	21.7% ± 1.9	0.1% ± 0.2
92.1	223	14.4% ± 2.7	0.2% ± 0.2
UW-1	66.33	12% ± 4.8	0% ± 0
SP6.5	266	3% ± 1.5	0% ± 0

## Discussion

It is known that protein tyrosine kinases (PTK) have an important role in cellular mechanisms, such as differentiation, proliferation and regulatory mechanisms, as well as in signal transduction. C-kit is one of these PTK, which is expressed in a wide variety of human malignancies [16] including chronic and acute myelogenous leukemia [6], GIST [7], mastocytosis [17], small cell lung carcinoma [18], chromophobe renal cell carcinoma [19], cutaneous [20] and UM [16]. As c-kit is expressed in normal interstitial cells of Cajal, the progenitor cell of GIST [7], the present article studies the expression of c-kit in uveal melanomas, as normal choroidal melanocytes do express this marker [21].

We demonstrated that more than 75% of UM from our series are positive for c-kit. This finding, per se, supports the idea of a clinical trial of imatinib mesylate for UM, especially in metastatic cases. Once metastatic disease is detected, no effective method of systemic therapy has been identified [3]. Moreover, not 100% of GIST is positive for c-kit. In fact, 6% of GIST are c-kit negative [22]. Before the imatinib mesylate "era", metastatic GIST had a median survival times ranging between 10–20 months [23]. Nowadays, imatinib mesylate controls tumor growth in up to 85% of advanced GIST [24], with 90% of acceptable toxicity [25].

In cutaneous melanoma, c-kit is strongly expressed in radial growth phase, and weak or no expression is seen in vertical growth phase and metastatic disease [26]. Therefore, in cutaneous melanomas c-kit expression appears to be related with stage of the disease. To further investigate a similar expression of the c-kit in UM, we observed the cell type (spindle and epithelioid) in which the c-kit was expressed, as it is well known that spindle cell type is less aggressive than epithelioid type [1]. None of the previous studies recorded the cell type in which the expression was occurring. Mouriaux *et al *[21] compared the c-kit expression with cell type tumor (Callendar's classification) as a correlation to prognostic factor, but did not study which cells were in fact expressing the receptor. In overall cases, including epithelioid, spindle and mixed tumors, 90.7% of the epithelioid cells was positive for c-kit, whereas 83.7% of the spindle cells expressed c-kit. This finding suggests a different role of c-kit expression in cutaneous and UMs. Therefore, c-kit can be used to differentiate primary UM from metastatic cutaneous melanomas to the uveal tract [27]. As in UM, GIST can be categorized into spindle cell, epitheloid cell and mixed cell type, although the prognostic relevance of cell type in GIST seems to be limited [7]. Like in UM showed by the present study, the expression of c-kit seems to be equally distributed among epithelioid and spindle cell morphology GISTs [28].

Pache *et al *[29] and Mouriaux *et al *[21] found a membrane pattern of immunoreactivity in all their positive cases (n = 72, 87% of all UM, and n = 43, 75% of all UM, respectively). The last suggested that cytoplasmatic immunoreactivity would be due to a non-mature c-kit rather than an internalized or truncated form, and the membrane staining could correspond to the c-kit active form. All-Ericsson *et al *[16] considered positive all tumors expressing c-kit, regardless of the staining location, meaning cell membrane or cytoplasmatic (n = 84, 63% of all UM). The present study demonstrated that all cases with high c-kit expression showed both cytoplasmatic and cell-membrane staining. Regarding the cases with low expression, 100% had cytoplasmatic expression, but only 30.4% had cell-membrane pattern of stain. In GISTs, the c-kit expression is diffuse strong cytoplasmatic and up to 50% of the cases show cytoplasmatic dot-like (so-called "golgi pattern") staining [7]. Moreover, there are no studies concerning important differences between cytoplasmatic and membranous staining in GIST. Therefore, we hypothesize that all cases expressing c-kit, cytoplasmatic or membranous, should be considered c-kit positive.

GIST is a sarcoma arising from the interstitial cells of Cajal, harbouring mutation of c-kit. Mutations are detected in approximately 71% of tumors, the majority (over 60%) involving exon 11, and less exons 9 and 13 [28]. Choroidal melanoma does not have alterations of exons 2, 8, 9, 11, 13 and 17 [29]. However, imatinib mesylate selectively inhibits not only c-kit, but also other tyrosine kinases such as Bcr-Abl and platelet-derived growth factor (PDGF) receptor [30]. We could demonstrate that the decrease of proliferation of UM cells with imatinib mesylate was very significant, in the 4 UM cell lines tested and in the human uveal transformed melanocyte cell line, compared to the control group. Other studies also support that imatinib mesylate can decrease UM cells proliferation rates [8]. The mechanism that c-kit would interfere in UM proliferation could be other than c-kit mutation, but further studies are necessary to investigate this hypothesis.

The concentration of imatinib mesylate used for the *in vitro *studies was 10 μM. This concentration is equivalent to the highest drug concentration achieved in the blood of patients receiving 1000 mg/day of imatinib mesylate, the maximum tolerated dose reported by clinical trials [14]. At that dose, the blood concentration of imatinib mesylate ranged from 6 to 10 μM [14]. According to clinical trials, the current treatment for GIST is 800 mg/day of imatinib mesylate [25]. All-Ericsson *et al *[16] demonstrated that concentrations of 10 μM of imatinib mesylate could inhibit the proliferation of 5 UM cell lines in 50% (2 of them, OCM-1 and 92.1, were also studied in this article). The different concentrations of imatinib mesylate tested had different responses according to the cell line. Pache *et al *[29] also had the same conclusion. Moreover, the last demonstrated that imatinib mesylate does not influence the proliferation of normal uveal melanocytes. The human uveal transformed melanocyte cell line UW-1 studied in the present article demonstrated a significant decrease in proliferation and invasion rates when treated with imatinib mesylate. UW-1 was originally derived from uveal melanocytes, and transformed into malignant melanoma cells throughout culture. We hypothesize that imatinib mesylate could act in a more general pathway than c-kit receptor, as it does inhibit UW-1 proliferation and invasion rates.

The effect on invasion of UM cells in response to imatinib mesylate has never been published before. Tumor cells must possess invasive abilities in order for metastasis to occur. Due to the lack of lymphatics in the eye, uveal melanoma cells must leave the primary tumor via hematogenous dissemination, with metastasis almost exclusively occurring in the liver [2]. Our study demonstrated that imatinib mesylate markedly reduced the invasiveness of all cell lines tested. The invasion assay is important to show the ability of cells to invade a basement membrane, simulating the escape of cells from the primary tumor, as well as the implantation of cells at the site of the metastasis. The use of artificial basement membrane can study the invasive response of cells to drugs by counting the amount of cells that invade the matrigel layer. A drug that can inhibit or reduce the invasiveness ability of the UM cells could be beneficial, as most of the UM are nowadays treated conservatively [31]. Decreasing the invasiveness of the tumoral cells, the drug would also decrease the ability of implantation of cells at the site of metastasis. Therefore, imatinib mesylate would be beneficial not only for UM patients that already developed metastasis, but also for patients without any sign of metastatic disease.

## Conclusion

We could demonstrate that primary choroidal melanomas express c-kit and imatinib mesylate decrease the proliferation rate and invasiveness of uveal melanoma cells *in vitro*. Therefore, our data supports a clinical trial for studying imatinib mesylate in uveal melanoma.
